# Bio-Inspired Microstructured Poly(vinylidene fluoride-co-hexafluoropropylene) Films Incorporated with Silver Nanoparticles for Antibacterial Applications

**DOI:** 10.3390/polym18101212

**Published:** 2026-05-16

**Authors:** Quang Hung Nguyen, Tien Thanh Nguyen, Zaki S. Saldi, Arief S. Budiman, Christian Harito, Monica Dwi Hartanti, Avinash Baji, Vi Khanh Truong

**Affiliations:** 1 Department of Engineering, La Trobe University, Bundoora, VIC 3086, Australia; 21456879@students.latrobe.edu.au; 2 Biomedical Nanoengineering Laboratory, College of Medicine and Public Health, Flinders University, Adelaide, SA 5042, Australia; nguy1184@flinders.edu.au (T.T.N.); vikhanh.truong@flinders.edu.au (V.K.T.); 3 Department of Product Design, Universitas Pembangunan Jaya, Tangerang Selatan 15413, Indonesia; zaki.saldi@upj.ac.id; 4 Industrial Engineering Department, BINUS Graduate Program—Master of Industrial Engineering, Bina Nusantara University, Jakarta 11480, Indonesia; arief.budiman@oit.edu (A.S.B.); christian.harito@binus.edu (C.H.); 5 Department of Manufacturing and Mechanical Engineering and Technology (MMET), Oregon Institute of Technology, Klamath Falls, OR 97601, USA; 6 Oregon Renewable Energy Center (OREC), Oregon Institute of Technology, Klamath Falls, OR 97601, USA; 7 Center for Biomedical Research, Organization for Health, National Research and Innovation Agency (BRIN), Jakarta 10340, Indonesia; mdhartanti@trisakti.ac.id; 8 Faculty of Medicine, Universitas Trisakti, Jakarta 11440, Indonesia

**Keywords:** *S. aureus*, *E. coli*, silver nanoparticles, antimicrobial, antibacterial material, microstructure, bio-inspired

## Abstract

In this study, poly(vinylidene fluoride-co-hexafluoropropylene) (PVDF-HFP) films embedded with silver nanoparticles were fabricated to investigate their antibacterial performance against *Escherichia coli* (*E. coli*) and *Staphylococcus aureus* (*S. aureus*). Inspired by the nanoscale topographies of natural antibacterial surfaces, such as dragonfly and cicada wings, microstructured pillars were introduced onto the polymer surface to enhance its bactericidal activity by increasing the effective contact area. Surface morphology was characterised using scanning electron microscopy (SEM), including higher-magnification imaging of micropillar surfaces, while energy-dispersive X-ray spectroscopy confirmed the presence of silver. Higher-magnification SEM revealed nanoscale surface features on the micropillars, attributed to embedded or surface-associated silver nanoparticles. Antibacterial performance was evaluated using confocal laser scanning microscopy with live/dead staining. The PVDF-HFP/Ag films exhibited a significant reduction in bacterial viability, particularly against *S. aureus* (reducing viability to 0.6% ± 1.1%), while showing moderate activity against *E. coli* (41.0% ± 3.7% viability). While the fabricated micropillars (~5 µm) are larger than bacterial cells and unlikely to induce direct mechanical rupture, they increase surface interaction. To further investigate the theoretical antibacterial mechanism of scaled-down features, finite element analysis (FEA) was performed to model the mechanical interaction between bacterial cells and nanostructured pillars. The simulation results indicated localised stress concentrations that could compromise bacterial membrane integrity, suggesting a possible mechanobactericidal contribution if the microstructures are further reduced to the nanoscale, in addition to the primary biochemical effects of silver nanoparticles. FEA results do not aim to explain the experimentally observed antibacterial performance and should be interpreted only as a conceptual investigation. These findings demonstrate the potential of bio-inspired PVDF-HFP/Ag films as antibacterial materials for food packaging and related applications, subject to future comprehensive toxicity and quantitative microbiological evaluations.

## 1. Introduction

Antimicrobial resistance (AMR) is emerging as a major threat to global public health and is widely recognised as one of the most critical concerns worldwide. In 2019, AMR was associated with 4.95 million deaths globally, including 1.27 million deaths directly attributable to bacteria resistant to antimicrobial treatments [1]. Some of the leading pathogens responsible for human deaths due to antimicrobial resistance are Escherichia coli (*E. coli*), *Staphylococcus aureus* (*S. aureus*), *Klebsiella pneumoniae*, *Streptococcus pneumoniae*, *Acinetobacter baumannii*, and *Pseudomonas aeruginosa*. Among these, *E. coli* and *S. Aureus* are the two bacteria responsible for the most deaths associated with antimicrobial resistance [1]. These pathogens are commonly found in various foods, including processed foods [2,3,4].

The growing threat of antimicrobial resistance necessitates alternative solutions to conventional antibiotic treatments. Nature has long inspired antibacterial strategies, particularly through micro- and nanoscale surface structures that passively inhibit bacterial colonisation [5,6]. Nanoscale structures found on dragonflies and cicada wings [7] have been shown to mechanically disrupt bacterial cell membranes [6,7]. Pioneering work by Ivanova et al. demonstrated that high-aspect-ratio nanopillars on cicada wings physically stretch and rupture Pseudomonas aeruginosa cells solely through mechanical forces [8]. Similarly, synthetic analogues like biomimetic black silicon have shown extraordinary mechanobactericidal efficiency against both Gram-negative and Gram-positive bacteria [9]. This natural defence strategy has inspired biomimetic approaches in material science [10], where micro- and nanostructured surfaces are engineered to enhance antibacterial properties [5].

Inspired by these natural antibacterial materials, the aim of this study is to integrate silver nanoparticles into surface-textured polymeric surfaces. PVDF-HFP was selected as the polymer matrix due to its excellent thermal stability, chemical inertness, high mechanical strength, and favourable film-forming properties, making it a highly suitable candidate for durable applications such as food packaging and biomedical devices [11,12,13]. Inorganic materials, such as zinc (Zn), silver (Ag), and copper (Cu), in their micro- or nanoscale forms, have emerged as promising antiseptic agents due to their potent antibacterial properties [14,15,16]. Polymer matrices such as chitosan effectively disperse silver and copper nanoparticles, forming polymer nanocomposites that exhibit excellent antibacterial activity against bacteria such as *E. coli* and *S. aureus* [17]. In this study, we produced microstructures on the surfaces of poly(vinylidene fluoride-co-hexafluoropropylene) (PVDF-HFP) films incorporating silver nanoparticles. These microstructures are specifically designed to increase the films’ surface area, potentially enhancing their antibacterial performance. Silver nanoparticles, renowned for their broad-spectrum antibacterial properties, disrupt bacterial cell membranes and interfere with critical enzymatic functions [18,19,20], making them highly effective across diverse applications, including food safety.

The structured polymer matrix not only mimics nature’s ability to physically disrupt bacterial membranes but also serves as a stable, controlled-release platform for AgNPs, thereby enhancing their antimicrobial efficacy [21]. Compared to previously reported ZnO-based microstructured systems and other antimicrobial polymer films, the present study focuses on a silver nanoparticle-based approach with distinct antibacterial mechanisms. Unlike nanoscale bactericidal surfaces reported in literature, the microscale features investigated here (~5 µm) are not expected to induce direct mechanical rupture of bacterial cells but may contribute to enhanced surface interaction. While nanoscale structured surfaces have been shown to induce mechanobactericidal effects, the microscale features investigated in this study (~5 µm) are not expected to cause direct mechanical rupture. Instead, antibacterial activity is primarily governed by interactions between silver nanoparticles, with surface structuring playing a secondary role. An FEA (finite element analysis) model is used in the present study to assess and estimate the theoretical effect of scaling these pillars down to the nanoscale on the silver nanoparticle coating in killing bacteria.

## 2. Materials and Methods

Silver nitrate (AgNO_3_) and poly(vinylidene fluoride-co-hexafluoropropylene) (PVDF-HFP), with an average molecular weight of 450,000, were obtained from Sigma-Aldrich, North Ryde, NSW, Australia. Acetone (C_3_H_6_O) and dimethylformamide (DMF), both used as solvents for the solution preparation, were sourced from CSA Scientific, Gilman, SA, Australia. A PVDF-HFP film incorporating silver nanoparticles (PVDF-HFP/Ag film) was fabricated using the casting method on a commercial template with 5 µm holes.

### 2.1. Sample Preparation

**Figure 1:**
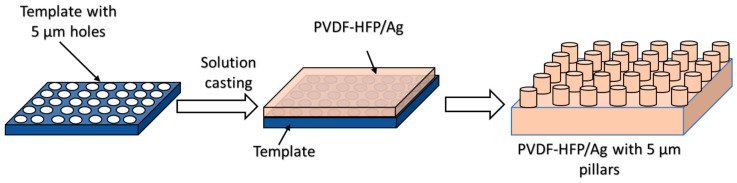
Schematic demonstrating the fabrication process used to prepare the samples. The PVDF-HFP/Ag solution is poured on the template consisting of 5 µm holes. Once the solvent is evaporated and the solution is solidified, the PVDF-HFP/Ag film is peeled off from the template to reveal 5 µm diameter pillars on its surface.

First, 0.1 g of silver nitrate (AgNO_3_) was added to 11 g of dimethylformamide (DMF) and stirred on a magnetic hot plate at 80 °C until the solution changed colour from colourless to brown. Next, 3.44 g of PVDF-HFP was dissolved in the AgNO_3_/DMF mixture, resulting in a PVDF-HFP concentration of 17.87 wt% and a final theoretical AgNP concentration of 0.52 wt%. Additionally, 4.71 g of acetone was added to achieve a DMF-to-acetone weight ratio of 70:30. The mixture was then stirred at 100 °C until the polymer was fully dissolved. Once the solution reached ambient temperature, it was cast onto a commercial template with 5-micron pores, as shown in Figure 1. The setup was left undisturbed for 24 h to allow the solvent to evaporate. The PVDF-HFP film incorporating silver nanoparticles (AgNPs) was then peeled off the template, revealing 5 µm diameter pillars on its surface. A neat PVDF-HFP/AgNP film was also prepared using a similar method. Here, the solution was cast onto a silica wafer to form a film without surface pillars, serving as an unpatterned control and thereby isolating the effect of the microstructure from the chemical effect of the AgNPs. A control sample was also prepared, which did not contain any silver nanoparticles or 5 µm pillars.

### 2.2. Surface Morphology Characterisation

The surface morphology of the prepared sample was investigated using ultra-high-resolution Schottky field-emission scanning electron microscopy (FESEM, Hitachi SU7000, Hitachi High-Tech Corporation, Tokyo, Japan) operated at an accelerating voltage of 10 kV. Before SEM imaging, the samples were sputter-coated with a thin layer of gold (18 mA, 60 s) to ensure conductivity. An atomic force microscope (AFM) (ezAFM, Nanomagnetics Instruments Ltd., Oxford, UK) was operated in tapping mode using a sharp tip cantilever (190 kHz resonant frequency, 10 nm tip radius) to further visualise and analyse the surface topography. Microstructural dimensions were quantified using ImageJ software (v1.54f) with statistical analysis based on at least ten independent measurements.

### 2.3. Antibacterial Properties

The antibacterial properties of the samples were investigated against *Staphylococcus aureus* (*S. aureus*) (ATCC 25923; American Type Culture Collection, Manassas, VA, USA) and *Escherichia coli* (*E. coli*) (ATCC 11303; American Type Culture Collection, Manassas, VA, USA). These bacterial strains were grown in the Lysogeny broth (LB) for a period of 18–24 h with conditions at 37 °C and shaking at 200 rpm. The final bacterial suspension was collected and diluted to obtain OD_600_ ≈ 0.001 (≈10^6^ CFUml^−1^) in LB medium for antibacterial inhibition assay. The antibacterial activity of the samples was investigated by testing on the surface. Briefly, samples were cut into small squares (0.5 cm × 0.5 cm) and soaked in 300 μL of the bacterial suspensions. While this static immersion method exposes the samples to bacteria throughout the bulk volume rather than exclusively on the surface, it provides a rigorous worst-case scenario for evaluating the combined efficacy of surface contact and localised silver ion release. These were placed in a 48-well plate and incubated again for 18 h at 37 °C without shaking. After incubation, the samples were carefully rinsed with PBS to prepare them for confocal laser scanning microscopy (CLSM, LSM 880 Fast Airyscan, Carl Zeiss Microscopy GmbH, Jena, Germany). The bacterial cells were examined using CLSM.

A second experiment was designed to evaluate the antibacterial and preservative properties of AgNPs. In this experiment, a grape was cut in half and placed on two different samples, one consisting of PVDF-HFP/Ag film and the other, a commercially available polyethylene (PE) food wrap. The grape was observed and compared at intervals of 0, 24, 48, and 72 h to assess the antibacterial and preservative effectiveness of the AgNPs. This evaluation aimed to determine their potential application in the food industry, not only as a solution for combating antibiotic-resistant bacteria such as *E. coli* and *S. aureus* but also as a superior alternative to conventional polyethylene (PE) film for food preservation.

### 2.4. Finite Element Simulations

To investigate the mechanism of bacterial inactivation using nanostructured surfaces, numerical simulations were performed using finite element analysis (FEA) within the ANSYS Mechanical 2025R2 environment. The objective was to evaluate the stress localisation and deformation of a bacterial cell envelope upon contact with an array of nanoscale pillars, a mechanism hypothesised to facilitate mechanobactericidal activity. The study adopted a linear static analysis to characterise the initial mechanical response of the bacterial wall. In this model, the cell envelope is treated as a passive mechanical structure, with regions of high stress concentration indicating potential sites of mechanical vulnerability and membrane damage. Although the fabricated pillars in this study are 5 µm in diameter, nanoscale pillar geometries were adopted in the simulation to represent the sharper surface features commonly associated with mechanobactericidal surfaces reported in the literature. This modelling approach enables evaluation of the stress localisation mechanism at the bacteria–pillar interface and provides insight into how reducing pillar dimensions may enhance antibacterial performance. Therefore, the simulation results should be interpreted as a mechanistic illustration of stress localisation rather than a direct quantitative representation of the experimentally fabricated micropillar geometry.

**Figure 2:**
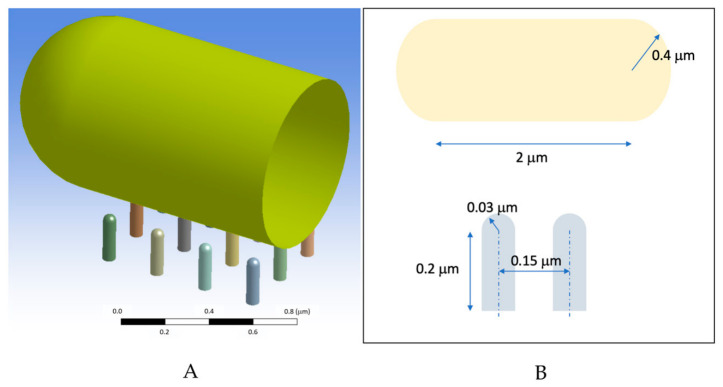
Schematic of the bacterial cell and the pillar. (**A**) Geometrical model of the bacterium on top of a 5 × 3 nanopillar array and (**B**) the dimensions of the bacterium and nanopillars (not to scale).

The physical model was represented by a simplified geometry of a capsule-shaped bacterium resting on top of a 5 × 3 nanopillar array (see Figure 2). To optimise computational efficiency, a half-symmetry model was employed (Figure 2). The bacterium itself was modelled as a thin, deformable shell with a uniform thickness of 1.5 nm. The bacterial wall mechanical properties were defined as homogeneous and isotropic, with a Young’s modulus of 25 MPa and a Poisson’s ratio of 0.2 [22]. While internal biological structures were not explicitly modelled, their mechanical influence was represented by applying a turgor pressure of 0.03 MPa to the inner surface of the shell, which effectively pushes the envelope against the pillar tips [22,23,24,25]. The nanopillars were modelled as solid elastic bodies of PVDF-HFP polymer, with a Young’s modulus of 500 MPa and a Poisson’s ratio of 0.40 [25]. Due to their significantly higher stiffness compared to the bacterial wall, these micropillars serve as rigid supports that induce localised indentation. The simulation’s boundary conditions included fully constraining the base of the micropillars to prevent rigid-body motion and applying symmetry conditions to the orthogonal plane. The system was discretised into 53,000 elements, using quadratic rectangular shell elements for the bacterial wall and quadratic tetrahedral solid elements for the micropillars. Mechanical interaction was managed through a surface-to-surface, penalty-based contact formulation with a friction coefficient of 0.2.

## 3. Results and Discussion

Nature has long served as an inspiration for the development of antibacterial materials [5]. Many organisms, such as dragonflies and cicadas, have evolved specialised surface structures with nanoscale topographies that passively inhibit bacterial growth by mechanically rupturing bacterial cell membranes, effectively preventing bacterial colonisation [26,27]. Similarly, the lotus leaf’s micro- and nanostructures create a superhydrophobic surface that discourages bacterial adhesion [28,29]. By replicating such micro- and nanostructured patterns and integrating antimicrobial agents, such as silver nanoparticles (AgNPs), innovative materials can be developed to combat bacterial contamination across various applications.

**Figure 3:**
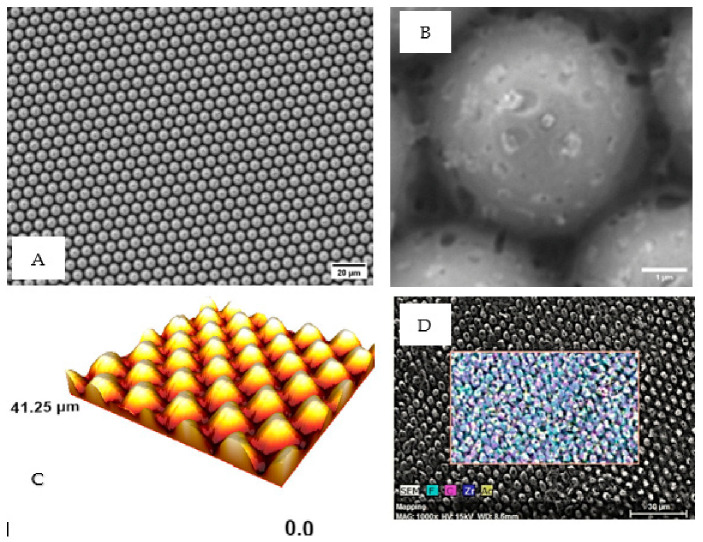
(**A**) SEM image showing the uniform distribution of micropillars (~5 µm diameter) on the PVDF-HFP/Ag film surface, (**B**) higher-magnification SEM image of a micropillar top surface. Some nanoscale features are visible in the figure and can be attributed to silver nanoparticles. These nanoparticles appear to be embedded within the polymer matrix or partially exposed to its surface, (**C**) AFM image demonstrating the surface morphology, and (**D**) SEM image with energy-dispersive X-ray spectroscopy (EDS) mapping.

Figure 3 shows the surface morphology of the solution-casted PVDF-HFP/Ag sample. It is evident from Figure 3 that casting PVDF-HFP/Ag solution onto the template yielded micropillars on the surface of the PVDF-HFP/Ag film. The micropillars are uniformly distributed on the film surface. The diameter of the micropillars is determined using ImageJ software to be ~5 µm, which closely matches the pore size on the template. A higher-magnification SEM image of the micropillar surface (Figure 3B) reveals nanoscale surface features distributed on the surface of the micropillar. These features appear as bright contrast regions and can be attributed to silver nanoparticles or clusters. It appears that these nanoscale features are embedded within or partially exposed on the polymer surface. Figure 3C also shows the surface morphology of the sample obtained using AFM. It can be confirmed that the micropillars are uniformly formed on the surface of the PVDF-HFP/Ag film.

**Figure 4:**
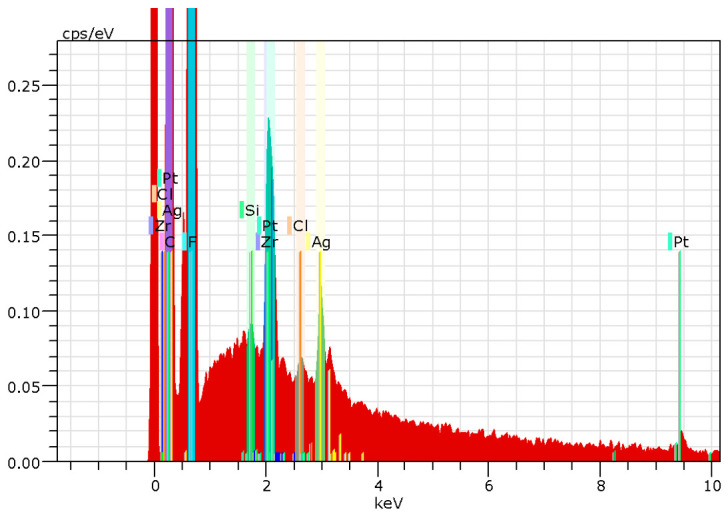
EDX spectrum of PVDF-HFP/Ag film.

Following this, energy-dispersive X-ray spectroscopy (EDS, Hitachi High-Tech Corporation, Tokyo, Japan) is used to confirm the formation of silver on the films. The tool is also used to investigate the sample’s elemental composition. Figure 3 shows the elemental map image of the sample. The EDS spectrum of the sample is also shown in Figure 4. The characteristic peaks that are associated with Ag, fluorine (F), and carbon (C) are visible in the spectra. The optical absorption band of Ag nanocrystallites associated with the peak at 3 keV is evident in the spectra. It is clear from the elemental map image and the spectra that there are strong signals from Ag and F atoms. This confirms the presence of silver within the PVDF-HFP film. However, it is acknowledged that EDS primarily confirms the presence of elements. Future studies utilising Transmission Electron Microscopy (TEM) and Inductively Coupled Plasma Mass Spectrometry (ICP-MS) will be necessary to fully characterise the nanoparticle size distribution and quantify the kinetics of silver ion release. It is important to distinguish between the experimentally fabricated micropillar structures (~5 µm) and the nanoscale geometries used in the finite element simulations. While the SEM results confirm the presence of microscale structures with nanoscale surface features, the FEA model investigates idealised nanoscale pillars to explore stress localisation mechanisms associated with mechanobactericidal activity. The simulation results presented in this article offer a framework for enhancing performance through nanoscale optimisation.

In the next step, the samples’ antibacterial activity is tested against *S. aureus* ATCC 25923 and *E. coli* ATCC 11303. The bacterial strains are grown in Lysogeny Broth (LB) at 37 °C with shaking at 200 rpm for 18 h. The final bacterial suspension was diluted in LB medium to an optical density of about 0.001 at 600 nm (OD_600_) for the antibacterial inhibition assay. The antibacterial activity of the samples is investigated by testing on the surface. Three different samples are tested: a control sample (neat PVDF-HFP film without any microstructures or Ag), a plain PVDF sample (PVDF-HFP film with surface structures), and a PVDF-HFP/Ag film with surface microstructures.

Briefly, samples are cut into small squares (0.5 cm × 0.5 cm) and soaked in 300 μL of bacterial suspensions. These are placed in a 48-well plate and incubated for an additional 18 h at 37 °C without shaking. After incubation, the samples are carefully rinsed with PBS to prepare them for confocal laser scanning microscopy (CLSM). The bacterial cells are examined using CLSM. To assess bacterial viability, we use the Live/Dead™ BacLight™ Bacterial Viability Kit (L7012; Invitrogen, Thermo Fisher Scientific, Waltham, MA, USA), which includes SYTO^®^ 9 and propidium iodide (PI). Next, 10 μL of the diluted Live/Dead™ BacLight™ dye solution is applied to the sample surface, followed by incubation in the dark for 15 min. SYTO^®^ 9 enters all bacterial cells, binding to their nucleic acids and emitting a green, fluorescent signal. In contrast, propidium iodide (PI) has a higher affinity for nucleic acids than SYTO^®^ 9 and only enters cells that are severely damaged or dead. A dual-emission filter in the CLSM is set up to detect live bacterial cells in green pixels (SYTO^®^ 9, Ex/Em 480/500 nm) and dead bacterial cells in red pixels (PI, Ex/Em 490/635 nm). Three surface images of each sample are randomly selected to analyse the ratio of viable to dead cells. Zen Black (v2.3) and ImageJ (v1.54f) software are used to analyse surface images, and the percentage of green and red pixels is calculated to measure the antibacterial activity.

**Figure 5:**
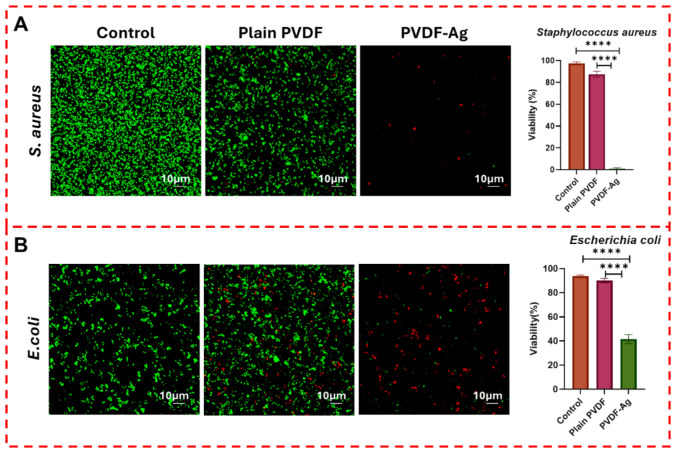
Visibility and bacterial viability of *S. aureus* (**A**) and *E. coli* (**B**) on control sample, plain PVDF, and PVDF-HFP/Ag film observed under CLSM. Scale bar 10 µm, *n* = 3. Data are presented as mean ± SD. **** indicates statistically significant differences (*p* < 0.0001).

Figure 5 shows the antibacterial properties of the samples. The antibacterial properties are assessed using the Live/Dead™ BacLight™ Bacterial Viability kit, which stains bacterial cells for observation using CLSM. In Figure 5A, *S. aureus* is found to be present in the control sample and the plain PVDF sample. They are densely populated on the plain PVDF film, with 87.4% ± 2.8% of bacteria appearing viable. This high percentage of viable cells indicates that plain PVDF lacks inherent antibacterial properties and is ineffective in preventing the survival of *S. aureus*. The densely populated bacterial communities on the plain PVDF film suggest that this material provides a favourable environment for bacterial adhesion and growth. In contrast, *S. aureus* on the PVDF-HFP/Ag film exhibited significantly lower viability of around 0.6% ± 1.1% (*p* < 0.00001), indicating a significant reduction in the bacterial population. It is important to note that while CLSM image-based pixel analysis provides a strong visual indicator of surface viability, it is inherently limited by the selected field of view. Future quantitative spectroscopic studies, such as Colony-Forming Unit (CFU) counting or Enzyme-Linked Immunosorbent Assays (ELISAs), are recommended to confirm bulk bactericidal efficiency.

The nearly complete eradication of *S. aureus* on the PVDF-HFP/Ag film suggests that the presence of silver nanoparticles plays a crucial role in disrupting bacterial cell function, leading to cell death. While the presence of microstructures may increase effective surface area and promote bacterial contact, this contribution is not independently quantified in the present study. Therefore, the antibacterial performance is primarily attributed to the biochemical activity of silver nanoparticles. Silver has long been known for its broad-spectrum antibacterial properties, primarily due to its ability to bind to bacterial cell membranes, disrupt membrane permeability, and interfere with cellular processes such as DNA replication and protein synthesis [30,31]. The results align with the existing literature on the antibacterial effects of silver-based materials. Studies have shown that silver nanoparticles can penetrate bacterial cell walls, causing structural damage and triggering the release of reactive oxygen species (ROS), which can further compromise bacterial viability [30,31]. In the case of *S. aureus*, the combination of these mechanisms likely contributed to the sharp decrease in viable cells observed on the PVDF-HFP/Ag film. Additionally, silver nanoparticles are known to disrupt the electron transport chain in bacteria [32], which could lead to energy depletion [33] and eventual cell death [33]. This multilevel attack on bacterial cells makes silver an effective agent against Gram-positive bacteria like *S. aureus*. These results reveal that the surfaces of the PVDF-HFP/Ag film exhibit excellent ability to kill and inhibit *S. aureus* (Gram-positive).

**Figure 6:**
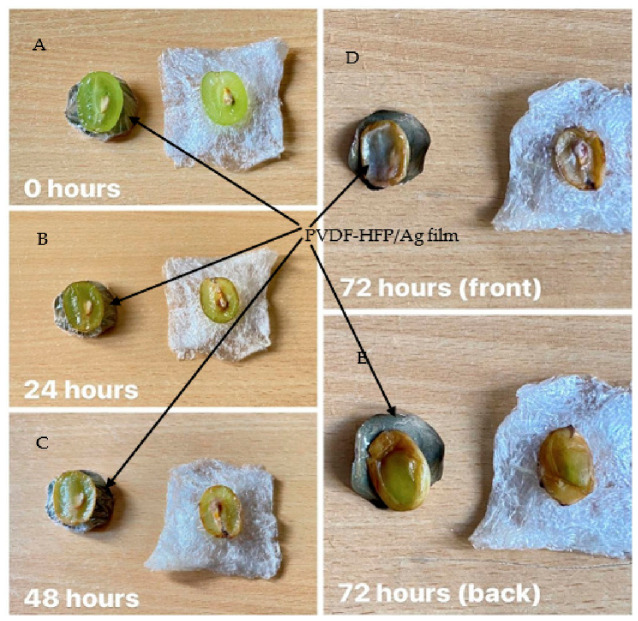
Visual comparison of grapes placed on microstructured PVDF-HFP/Ag film (left) and commercial PE food wrap (right) over time. (**A**) 0 h, (**B**) 24 h, (**C**) 48 h, (**D**) 72 h front view, and (**E**) 72 h back view. The images serve as a qualitative demonstration of the potential effect of the films on grape surface condition. No quantitative microbiological or physicochemical measurements were performed.

The antibacterial effectiveness of the film samples against Gram-negative bacteria is evaluated using *E. coli* as the pathogen (Figure 6B). Plain PVDF showed almost no ability to eliminate *E. coli*, with approximately 90% ± 1.8% of the bacteria surviving on its surface, nearly identical to the control. This result is consistent with findings for *S. aureus*, reinforcing the notion that plain PVDF lacks the antibacterial properties needed to inhibit bacterial growth. In comparison with plain PVDF, the inclusion of silver in the PVDF matrix significantly enhanced its antibacterial properties, reducing bacterial viability to 41% ± 3.7% (*p* < 0.00001). This finding indicates that the PVDF-HFP/Ag polymer film is highly effective against bacterial cells, as evidenced by reduced bacterial viability. It was particularly potent in eradicating and inhibiting the replication of Gram-positive bacteria, such as *S. aureus*. However, it is less effective against the Gram-negative bacterium *E. coli*. Figure 5 also shows the effect of the surface micropillar array/structures separately from the presence of silver. It is evident that the surface micropillar array/structures reduce the visibility and bacterial viability of both *S. aureus* and *E. coli* (about a 10–15% reduction in bacterial viability from control to plain PVDF samples), although the effect of the silver coating is more substantial. Because the 5 µm pillars are significantly larger than the bacterial cells, this modest reduction is likely not due to direct membrane piercing, which requires nanoscale sharpness but rather due to an increase in effective surface contact area, which may restrict bacterial motility and alter local biochemical signalling paths. This finding suggests the possibility of pure polymer surface nanostructures, which could be engineered to exhibit superior mechanobactericidal action. Such a possibility is appealing for developing chemical-free antibacterial materials useful in food packaging and in invasive biomedical devices, such as catheters, that require antimicrobial surfaces.

The difference in antibacterial efficacy between Gram-positive and Gram-negative bacteria can be attributed to the structural differences in their cell walls. The structural differences between Gram-positive and Gram-negative bacteria fundamentally alter their interactions with the material. The lower antibacterial efficiency determined for *E. coli* compared with *S. aureus* can be attributed to the presence of the outer membrane in Gram-negative bacteria, which acts as an additional barrier that limits the penetration of silver ions [30,34,35,36]. Furthermore, the mechanisms of antibiotic and nanometal resistance differ significantly between these groups; relying solely on surface morphology and elemental composition provides only a partial understanding of the complex biochemical signalling and stress responses occurring at the bacterial–material interface. This structural difference is likely the reason why *E. coli* demonstrated a higher survival rate on the PVDF-HFP/Ag film compared to *S. aureus*. Furthermore, the outer membrane of Gram-negative bacteria contains efflux pumps and other defensive mechanisms that can actively expel harmful substances, including silver nanoparticles, from the cell [37]. These protective measures can reduce the overall effectiveness of silver-based antibacterial agents when targeting Gram-negative bacteria [38]. Despite these challenges, the 41% reduction in *E. coli* viability on the PVDF-HFP/Ag film still represents a significant antibacterial effect, suggesting that silver nanoparticles can overcome some of these defences, albeit less efficiently than with Gram-positive bacteria.

Following this, the potential of using the samples for food packaging is explored. The experiment involved cutting a grape in half and placing each half on two different materials, viz. PVDF-HFP/Ag film and commercial polyethene (PE) food wrap. Both halves were wrapped using commercial PE food wrap. Images of both samples were taken at 0, 24, 48, and 72 h. At the 72 h mark, the wrapping was removed, and each grape half was imaged from both the front and back to assess the food-preservation properties of the PVDF-HFP/Ag film.

It is evident from Figure 6 that at 0 h, both grape halves appeared fresh. The differences between the two halves were apparent after the 24 h interval. The grape half placed on the commercial PE food wrap deteriorated faster than the half placed on the PVDF-HFP/Ag film. Signs of deterioration in the former included darker colouration and the appearance of black streaks, particularly visible on the stem and seeds by the end of the 48 h interval. At the 72 h mark, both grape halves showed signs of rotting, but the one placed on the commercial PE food wrap showed more severe withering. Examination of the reverse side revealed that the grape half on the commercial PE food wrap was more severely dehydrated and decayed, with numerous black spots around the edges. In contrast, the grape half placed on the PVDF-HFP/Ag film retained a relatively fresh green colour with minimal signs of deterioration. These results provide a qualitative demonstration of the potential food preservation capability of the PVDF-HFP/Ag films. We note that this was a preliminary, unrepeated qualitative observation lacking strict environmental controls. Because the commercial PE wrap was used as an over-wrap for both samples, it may have introduced confounding variables. Future rigorous testing must include standard microbiological swabbing of the food surface, unwrapped glass controls to assess natural spoilage, and, critically, comprehensive mammalian cytotoxicity and leaching assays to ensure the material is safe for direct food contact [39].

**Figure 7:**
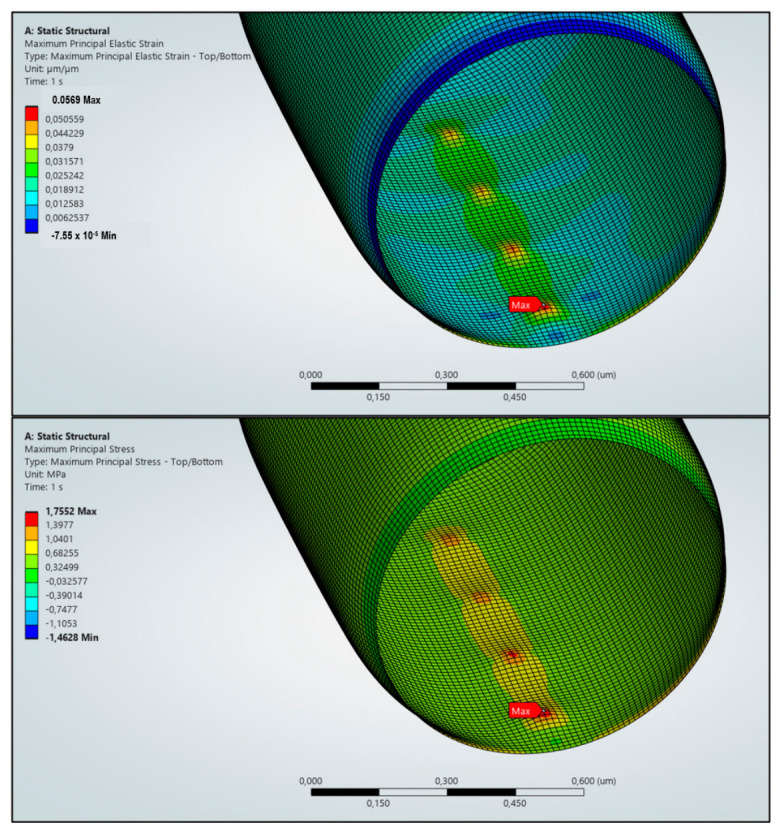
The maximum principal stress field (**top**) and the maximum principal strain field (**bottom**) at the bacteria wall.

We also performed the simulation to explore the theoretical limits of the structured films at smaller scales. It is important to distinguish between the experimentally fabricated micropillar structures (~5 µm) and the nanoscale geometries used in the finite element simulations. While the SEM observations confirm the presence of microscale structures with nanoscale surface features, the FEA model investigates idealised nanoscale pillars to explore stress localisation mechanisms associated with mechanobactericidal activity. Therefore, the experimental results validate the structural platform, while the simulations provide a conceptual framework for potential performance enhancement through nanoscale optimisation. While the experimental pillars are larger (~5 µm), the nanoscale model illustrates the stress localisation mechanism that would occur if the pillar dimensions were reduced to scales comparable to those found in natural bactericidal surfaces. The simulation results, illustrated in Figure 7, provided the distributions of maximum principal stress and strain along the bacterial wall. As the internal turgor pressure forced the envelope to conform to the curvature of the micropillar tips, localised indentation occurred, leading to a rise in mechanical load at the contact points. The highest stress levels are observed at the pillars closest to the central symmetry plane, reaching a maximum principal stress of approximately 1.76 MPa and a maximum principal strain of 0.0569. As this stress value far exceeds the typical yield strength range of 50–500 kPa associated with bacterial walls [22,23,24], it can be implied that the induced physical deformation is sufficient to compromise the structural integrity of the cell.

However, these findings must be viewed through the lens of the linear elastic approach used in the current study. As the model assumes small strains and reversible material behaviour, it does not explicitly capture irreversible phenomena such as plastic deformation, time-dependent viscoelasticity, and the actual membrane rupture. Furthermore, regions of high stress do not automatically equate to bacterial death; a more comprehensive future analysis must model the effective contact area and the thermodynamic energy required for membrane penetration. Therefore, subsequent steps of the simulation studies will focus on expanding the model to include nonlinear material properties and explicit damage criteria. The FEA simulation results support the concept of developing purely polymer-based mechanobactericidal surfaces. In polymers, nanostructures such as nanowires and nanotubes often exhibit enhanced stiffness and strength due to their constrained volumes. Notably, the current model shown in Figure 7 assumes a relatively moderate Young’s modulus of 0.5 GPa, whereas PVDF-HFP has been reported to possess a modulus in the range of 2.5–3.5 GPa. The strength and stiffness of PVDF nanopillar arrays can potentially be enhanced through nanoscale engineering methods, such as laser-induced crystallisation or plasma surface treatment [40]. This advancement allows for the development of purely polymer-based mechanobactericidal surfaces. As the tip radius of the pillars becomes sharper, the resulting stress concentration increases, potentially significantly enhancing their bactericidal effectiveness.

It should also be noted that the current experimental results are based on pillars with diameters of approximately 5 µm, whereas bacterial cells typically have dimensions on the order of a few micrometres. This size mismatch may partly explain the relatively modest reduction in bacterial viability (approximately 10–15%) observed when comparing neat PVDF-HFP surfaces with PVDF-HFP surfaces containing micropillar arrays. The present simulations further suggest that substantially higher bactericidal efficiency could be achieved by reducing the pillar arrays to the nanometre scale. It is important to emphasise that the nanoscale pillar geometry used in the finite element analysis does not represent the experimentally fabricated micropillar structures (~5 µm). The FEA results are not intended to explain the experimental antibacterial performance and should be interpreted solely as a conceptual exploration of potential mechanistic behaviour at reduced length scales.

## 4. Conclusions

This study highlights the antibacterial potential of microstructured PVDF-HFP/Ag films, particularly against *Staphylococcus aureus* (0.6% viability) compared to *Escherichia coli* (41.0% viability). The confocal laser scanning microscopy (CLSM) results underscored the potent bactericidal properties of AgNPs. In contrast, plain PVDF films exhibited no meaningful antibacterial effect, indicating that the 5 µm micropillars alone are insufficient for complete bacterial eradication, though they modestly contribute by increasing the active surface area for silver interaction. The discrepancy in efficacy is likely due to the structural differences in bacterial cell walls, with the outer membrane of Gram-negative bacteria acting as a protective barrier. Furthermore, our finite element modelling of nanoscale pillars demonstrated that reducing the surface feature size to the nanoscale could induce localised mechanical stress exceeding the bacterial cell wall yield strength, pointing toward a synergistic mechanobactericidal and biochemical mechanism for future material iterations. Finally, preliminary qualitative tests suggest utility in food preservation, though rigorous quantitative toxicity, silver leaching, and standardised microbiological studies are mandatory prior to practical application. Future work will focus on comprehensive nanoparticle characterisation, quantitative antibacterial assays, and toxicity evaluation to establish the practical applicability of the developed films.

## Data Availability

The original contributions presented in this study are included in the article. Further inquiries can be directed to the corresponding author.
